# Comparison of muscle synergies for running between different foot strike patterns

**DOI:** 10.1371/journal.pone.0171535

**Published:** 2017-02-03

**Authors:** Koji Nishida, Shota Hagio, Benio Kibushi, Toshio Moritani, Motoki Kouzaki

**Affiliations:** 1 Laboratory of Neurophysiology, Graduate School of Human and Environmental Studies, Kyoto University, Sakyo-ku, Kyoto, Japan; 2 Research Fellow of the Japan Society for the Promotion of Science, Chiyoda-ku, Tokyo, Japan; 3 Laboratory of Applied Physiology, Graduate School of Human and Environmental Studies, Kyoto University, Sakyo-ku, Kyoto, Japan; West Virginia University School of Medicine, UNITED STATES

## Abstract

It is well known that humans run with a fore-foot strike (FFS), a mid-foot strike (MFS) or a rear-foot strike (RFS). A modular neural control mechanism of human walking and running has been discussed in terms of muscle synergies. However, the neural control mechanisms for different foot strike patterns during running have been overlooked even though kinetic and kinematic differences between different foot strike patterns have been reported. Thus, we examined the differences in the neural control mechanisms of human running between FFS and RFS by comparing the muscle synergies extracted from each foot strike pattern during running. Muscle synergies were extracted using non-negative matrix factorization with electromyogram activity recorded bilaterally from 12 limb and trunk muscles in ten male subjects during FFS and RFS running at different speeds (5–15 km/h). Six muscle synergies were extracted from all conditions, and each synergy had a specific function and a single main peak of activity in a cycle. The six muscle synergies were similar between FFS and RFS as well as across subjects and speeds. However, some muscle weightings showed significant differences between FFS and RFS, especially the weightings of the tibialis anterior of the landing leg in synergies activated just before touchdown. The activation patterns of the synergies were also different for each foot strike pattern in terms of the timing, duration, and magnitude of the main peak of activity. These results suggest that the central nervous system controls running by sending a sequence of signals to six muscle synergies. Furthermore, a change in the foot strike pattern is accomplished by modulating the timing, duration and magnitude of the muscle synergy activity and by selectively activating other muscle synergies or subsets of the muscle synergies.

## Introduction

Runners are broadly categorized into three groups according to their foot strike pattern [1–8]. These patterns include a fore-foot strike (FFS), in which the ball of foot lands before the heel; a mid-foot strike (MFS), in which the heel and ball of the foot land simultaneously; and a rear-foot strike (RFS), in which the heel lands first. In particular, the differences between the FFS and RFS have been studied in terms of ground reaction forces [1], knee loading [2], and running economy [3]. Yong et al. [4] showed a difference in muscle activity between habitual FFS and RFS runners. According to their findings, FFS runners showed significantly lower activity in the tibialis anterior during the terminal swing phase. In contrast, the medial and lateral gastrocnemius showed greater activity in the terminal swing phase in FFS runners. The same differences in muscle activity were also reported when habitual RFS runners ran with their natural RFS pattern and a FFS pattern [5]. However, those studies focused on the differences in the final output parameters and did not reveal differences in the neural control mechanisms that produced the different outputs.

The neural control mechanisms of human running are still unclear. Because humans and other species have a large number of muscles, motor control would be redundant if the central nervous system (CNS) individually issued commands to each muscle. To solve this redundancy problem, previous studies have suggested a modular neural control mechanism referred to as muscle synergies [9–16]. Assuming the existence of muscle synergies, the CNS issues commands to a small number of muscle synergies to achieve movements. Human running has also been discussed in terms of muscle synergies [17,18]. Cappellini et al. [17] suggested that both human walking and running might be controlled by a sequence of five temporal activation components. Hagio et al. [18] showed that the gait transition between walking and running was controlled by approximately nine muscle synergies. In those studies, the authors focused on comparing muscle synergies between walking and running. However, running includes different foot strike patterns [1–8], and there are likely changes in muscle synergies or in the activation patterns of muscle synergies that must occur to change foot strike patterns during running. Comparing the muscle synergies extracted during running with different foot strike patterns revealed the neural control mechanisms that modulate the activity of a large number of muscles to realize those running styles.

The main purpose of the present study was to compare muscle synergies for running with different foot strike patterns. To make a simple comparison, we classified running styles performed in the experiment into FFS and RFS running based on footswitch signals. We hypothesized that the results of the present study would reveal a modular neural control mechanism underlying human running and explain how the CNS controls running with different foot strike patterns.

## Materials and methods

### Subjects

Ten healthy male subjects (mean ± SD: age, 21–25 years; height, 1.71 ± 0.05 m; body mass, 62 ± 4 kg) volunteered for the experiments. They were asked whether they habitually run with FFS or RFS. Two subjects (Sub5 and Sub10) answered that they were habitual FFS runners, and the rest of the subjects answered that they were habitual RFS runners. The experimental procedures were conducted in accordance with the Declaration of Helsinki and were approved by the Local Ethics Committee of the Graduate School of Human and Environmental Studies, Kyoto University (26-H-23). The ten subjects were named as follows for convenience: Sub1, Sub2, Sub3, Sub4, Sub5, Sub6, Sub7, Sub8, Sub9, and Sub10.

### Experimental setup and tasks

The experiments were carried out on a treadmill (Adventure3 Plus, Horizon Fitness, Johnson Health Tech Japan Co., Tokyo, Japan). The running surface of the treadmill was 1.41 m long and 0.5 m wide [18]. Subjects ran on the treadmill at different speeds (5, 7, 9, 12, and 15 km/h). At each speed, they were asked to run with both FFS and RFS. Subjects ran with shoes on. In real time, foot strike patterns were confirmed by footswitches attached bilaterally to the fore and rear part of the shoe soles [18]. The front switch was located on 70% of the shoe-sole length from the rear edge and the rear switch was located on 15% of the shoe-sole length from the rear edge. We confirmed that subjects were running with FFS when front footswitches turned on first, and that they were running with RFS when rear footswitches turned on first. To verify foot strike patterns, we also calculated the foot strike angle (FSA) of the foot using kinematic data [19]. Before the recording session, subjects practiced for a few minutes running on the treadmill at different speeds with both FFS and RFS to adjust to those running styles.

### Data recording

Surface electromyogram (EMG) activity was recorded from the following 12 muscles bilaterally: medial gastrocnemius (MG), lateral gastrocnemius (LG), soleus (Sol), tibialis anterior (TA), vastus lateralis (VL), rectus femoris (RF), biceps femoris (long head, BF), tensor fascia latae (TFL), adductor longus (AL), gluteus medius (Gmed), gluteus maximus (Gmax), and erector spinae (ES). Bipolar Ag-AgCl electrodes, with a diameter of 10 mm and inter-electrode distance of 20 mm, were used. The electrode placement was carefully chosen to minimize crosstalk from adjacent muscles using a B-mode ultrasonic apparatus (α-6, Aloka, Tokyo, Japan) [20]. The common reference electrodes were placed on the right ankle and the wrists. The electrodes were connected to a pre-amplifier and differential amplifier (× 1000) with a bandwidth of 5–480 Hz (SX230, Biometrics Ltd., Newport, United Kingdom) [21].

Kinematic data was recorded bilaterally at 100 Hz by means of the three-dimensional optical motion capture system (OptiTrack V100, NaturalPoint Inc., Oregon, the United States) with eighteen cameras spaced around the treadmill. Infrared reflective markers were attached on each side of the subject to the skin overlying the following landmarks: temple, acromion, lateral condyle of the elbow, styloid process of the ulna, anterior superior iliac spine, posterior superior iliac spine, greater trochanter, lateral condyle of the knee, medial condyle of the knee, lateral malleolus, medial malleolus, heel, and toe. The markers were also attached to vertex, chin, and right blade bone.

The signals recorded from the EMG electrodes and foot switches were stored at the sampling frequency of 1000 Hz on the hard disk of a personal computer using a 16-bit analog-to-digital converter (PowerLab/16SP; AD Instruments, Sydney, Australia). Sampling of EMG, foot switches, and kinematic data were synchronized.

Recording was started after the subjects had been running on the treadmill for about half a minute to allow their movement to settle into a regular pattern. More than 30 gait cycles were recorded for each running condition.

### Data analysis

All analyses were performed with custom software written in Matlab (Mathworks, Natick, MA). The gait cycle was defined with respect to right leg movement and began when the right foot contacted the foot switch and initiated the corresponding signal. The front switch was used to indicate the start of the gait cycle in FFS running and the rear switch was used to indicate the start of the gait cycle in RFS running.

#### Foot strike angle

In order to verify that the subjects were in fact running with FFS or RFS, we calculated FSA [19] using kinematic data. FSA was defined as

$$\text{FSA}=∠{\text{AB}}_{\text{footstrike}}-∠{\text{AB}}_{\text{baseline}}$$

where ∠AB_footstrike_ was the angle at foot strike between vector AB, which was parallel to heel-toe segment, and horizontal axis in the sagittal plane, and ∠AB_baseline_ was the averaged angle of the foot during the periods when both rear and front footswitches were on. A positive FSA indicated dorsiflexion.

#### Data preprocessing

The EMG data recorded for each condition, which included 30 consecutive gait cycles, were high-pass filtered at 40 Hz using a zero-phase-lag 4th-order Butterworth filter, full-wave rectified, and low-pass filtered at 10 Hz [17,22]. The filtered data were time-interpolated over a time base with 200 points [17,18,22,23] for individual gait cycles (cubic spline interpolation). The calculated data for each condition were arranged into a data matrix with 24 rows (24 muscles) and 6000 columns (200 points/cycle × 30 cycles). The amplitudes of the EMG waveforms in a data matrix were normalized by the maximum value in 10 data matrices (5 speeds × 2 foot strike patterns) for the corresponding subject so that all muscle scales ranged from 0 to 1 and also normalized by the standard deviation values for the corresponding muscles in the data matrix to have unit variance and thus ensure that the activity in all muscles was equally weighed [18]. After being low-pass filtered and time-interpolated, a small fraction of the EMG samples (0.01% and 0.005% of total trials, respectively) assumed negative values. However, to use the non-negative matrix factorization (NMF), all negative values were set to zero as NMF is constrained to positive values in the data matrix [16].

#### Extraction of muscle synergies

Muscle synergies were extracted from each data matrix of EMG recordings using the NMF [9–16,18,22,24] algorithm. NMF assumes that a muscle activation pattern *M* in a given time period is composed of a linear combination of a few muscle synergies *W*_*i*_ that are each recruited by a synergy recruitment coefficient *C*_*i*_. Therefore, a particular muscle activation pattern *M* in a task would be represented by

$$M=\sum_{i=1}^{N_{syn}}W_{i}C_{i}+\varepsilon \;(W_{i}\geq 0,\;C_{i}\geq 0)$$

where we specify the relative contributions of the muscles involved in synergy *i*. Each muscle synergy has a fixed composition *W*_*i*_ (a row vector of 24 elements) and is multiplied by a scalar recruitment coefficient *C*_*i*_ (a column vector of 6000 elements), which changes over time. *ε* is residual. The detailed extraction procedure is as follows. (1) For each *N*_*syn*_ (number of synergies), *W* and *C* matrices were initialized randomly and updated 3000 times to sufficiently minimize the residual error *ε* between the original data matrix and the reconstructed data matrix obtained by multiplying *W* and *C*. (2) To eliminate the influence of initial value dependence, ten runs of the NMF algorithm were performed, and the best solution that showed the highest variance accounted for (VAF) value (see the next paragraph) was selected [16]. (3) The synergy weighting and activation coefficient matrices were normalized such that the individual weighting vector was the unit vector [18].

#### Selection of the number of synergies

The goodness of fit of the data reconstruction using each number of muscle synergies was quantified by the VAF, which was defined as 100 × (1 − SSE/SST), with SSE representing the sum of square residuals of the data reconstruction and SST representing the sum of the squared data. The number of synergies was determined by choosing the least number of synergies that could account for greater than 90% of the overall VAF and for greater than 75% of the VAF in each muscle. This threshold is the same as that used in a previous study about human walking [15].

**Fig 1:**
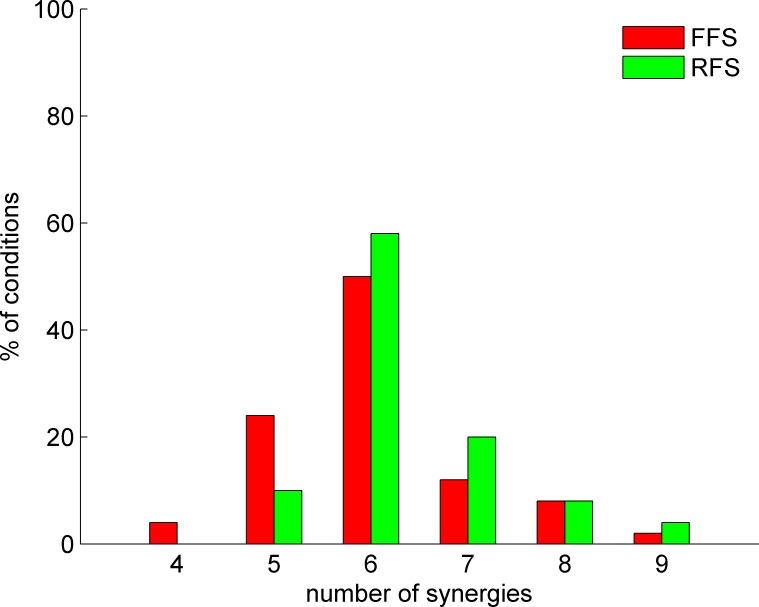
Histogram of the number of synergies determined by the variance accounted for (VAF) threshold criterion. The histogram incudes all speed tasks for all subjects for fore-foot strike (FFS) (red bars) and rear-foot strike (RFS) (green bars).

According to this threshold criterion, four to nine muscle synergies were extracted for all conditions. However, the EMG activity in most conditions was well accounted for by six synergies, and the variation seemed to be normally distributed (Fig 1). Hence, we set six as the conclusive number of synergies for all conditions. We again used the NMF algorithm in all conditions for the six synergies. The six synergies in an arbitrary subject were arranged in order according to the timing of the main peak of their activation patterns and then the six synergies in the other subjects were sorted based on the values of cosine similarity (see the next paragraph) with that of the arbitrary reference subject. The sorted six synergies were named in order as Syn1, Syn2, Syn3, Syn4, Syn5 and Syn6.

#### Muscle synergy comparison

To examine the similarity in weightings among the muscle synergies across conditions, we used a cosine similarity analysis [25]. In this analysis, when comparing two muscle synergies, the inner product of the two muscle synergy vectors was calculated. Because each weighting vector was normalized by its norm, the inner product of the two muscle synergy vectors represented the cosine of the angle between the vectors. Thus, an inner product closer to 1 indicated a greater similarity in the directions of the two vectors.

To compare muscle synergies extracted from FFS and RFS, we also examined the phase shifts and the differences in the duration and magnitude of the activation patterns. The phase shifts were quantified by comparing the timing of the main peaks of the activity. The main peak timings were transformed to radians ranging from -π to π for circular statistics in order to eliminate the influence of the peaks stepping over gait cycles. The duration of the activity was defined as the full-width at half-maximum (FWHM) of the main peak [17,22]. The magnitude of the activity was examined by calculating the root mean square (RMS) of the activity during the FWHM. Significant differences in muscle weightings, FWHM, and RMS between FFS and RFS were calculated using paired *t*-tests. Significant differences in phase shifts were assessed using the Watson-Williams test for circular data [22]. The significance of the differences was set at *p* < 0.05.

## Results

### Verification of foot strike patterns

**Fig 2:**
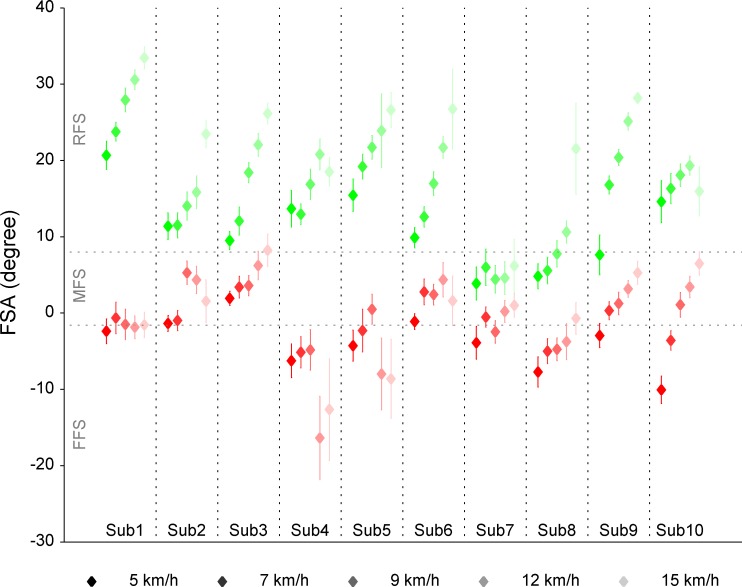
Foot strike angle (FSA). Diamonds and error bars represent mean FSAs and SDs over cycles. Reds for FFS tasks and greens for RFS tasks. Horizontal dotted gray lines represent 8.0° and -1.6°, respectively.

In the experiment, we confirmed foot strike patterns using footswitches in real time. However, this method has not been validated and it was insufficient to verify foot strike patterns. Thus we calculated FSA [19] using kinematic data. Following the criterion shown in the previous study [19], we classified foot strike patterns: FFS = FSA < −1.6°, RFS = FSA > 8.0°, and MFS = −1.6° < FSA < 8.0°. Fig 2 shows FSA in all tasks for every subject. According to the criterion, many foot strike patterns performed in FFS tasks were classified into MFS and some in RFS tasks were also classified into MFS. However, each subject showed larger FSA in RFS tasks than in FFS tasks. Furthermore, in all subjects, the front switch did turn on first in FFS tasks and the rear switch did turn on first in RFS tasks. This confirms the fact that the subjects were actually running with FFS in FFS tasks and with RFS in RFS tasks because the positions of the front and rear footswitches were within the areas in which the center of pressure at touchdown indicates FFS and RFS, respectively [19,26]. Note that we focused on revealing how the CNS coordinates muscle activities when it intends to change a foot strike pattern. Thus it is most important that subjects try to run with FFS or RFS in each task and it is not so essential that they run in fact with FFS or RFS according to the criterion shown in the previous study [19]. Therefore in this study, we determined that subjects were running with FFS or RFS based on footswitch signals.

### EMG waveforms

**Fig 3:**
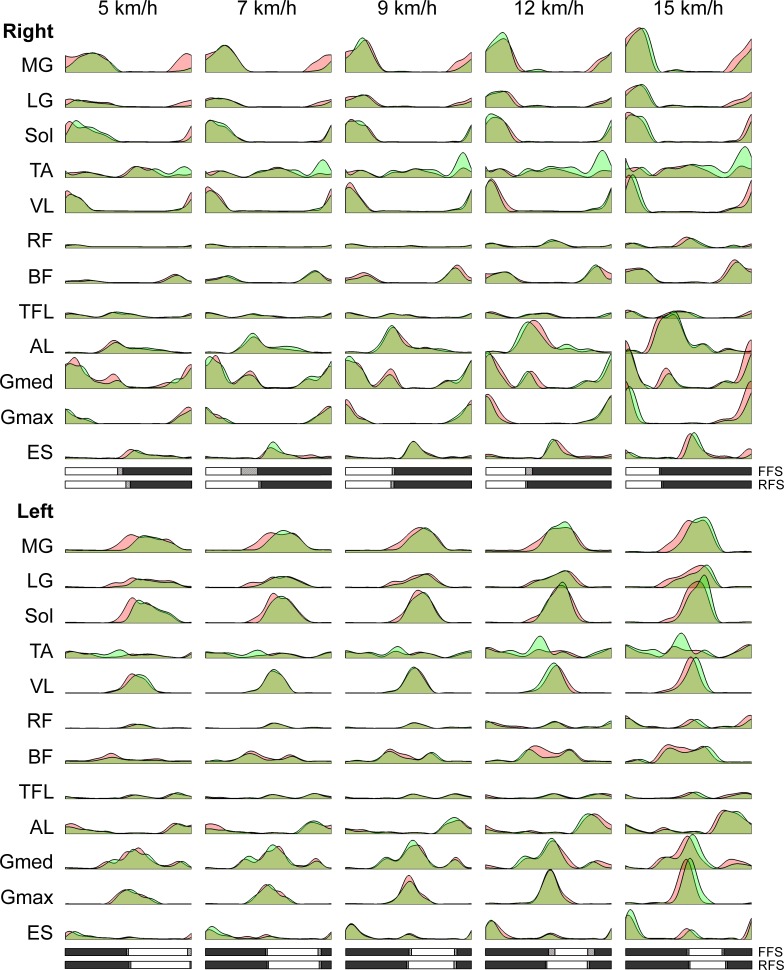
Muscle activity patterns for 24 muscles in FFS and RFS. Recorded electromyogram (EMG) activity was high-pass filtered (40 Hz), rectified, low-pass filtered (10 Hz), and time-interpolated (200 points). The red and green represent FFS and RFS, respectively. The activity in RFS was superimposed on the activity in FFS. Thus the dark green indicates the overlap between FFS and RFS. Bars at the bottom of the waveforms represent the stance and swing phases in one cycle (white: stance phase, gray: swing phase, shaded: mean ± SD of liftoff or touchdown timing over cycles). The upside bars for FFS and the downside bars for RFS. Shown here are medial gastrocnemius (MG), lateral gastrocnemius (LG), soleus (Sol), tibialis anterior (TA), vastus lateralis (VL), rectus femoris (RF), biceps femoris (long head, BF), tensor fascia latae (TFL), adductor longus (AL), gluteus medius (Gmed), gluteus maximus (Gmax), and erector spinae (ES) on the left and right sides.

The EMG activity for one cycle during running at various speeds in FFS and RFS are illustrated in Fig 3. The waveforms are similar to those observed in the previous study [17]. The amplitude of the EMG activity increased with an increase in speed. In almost all muscles, there is no determinant difference between FFS and RFS. However, TA showed higher activity in RFS during the terminal swing phase. On the other hand, the plantar flexors (MG, LG, and Sol) showed higher activity during terminal swing phase in FFS, indicating that their amplitudes around the touchdown were higher in FFS or that the onset of their activity was earlier in FFS.

### Similarity in muscle synergies

**Fig 4:**
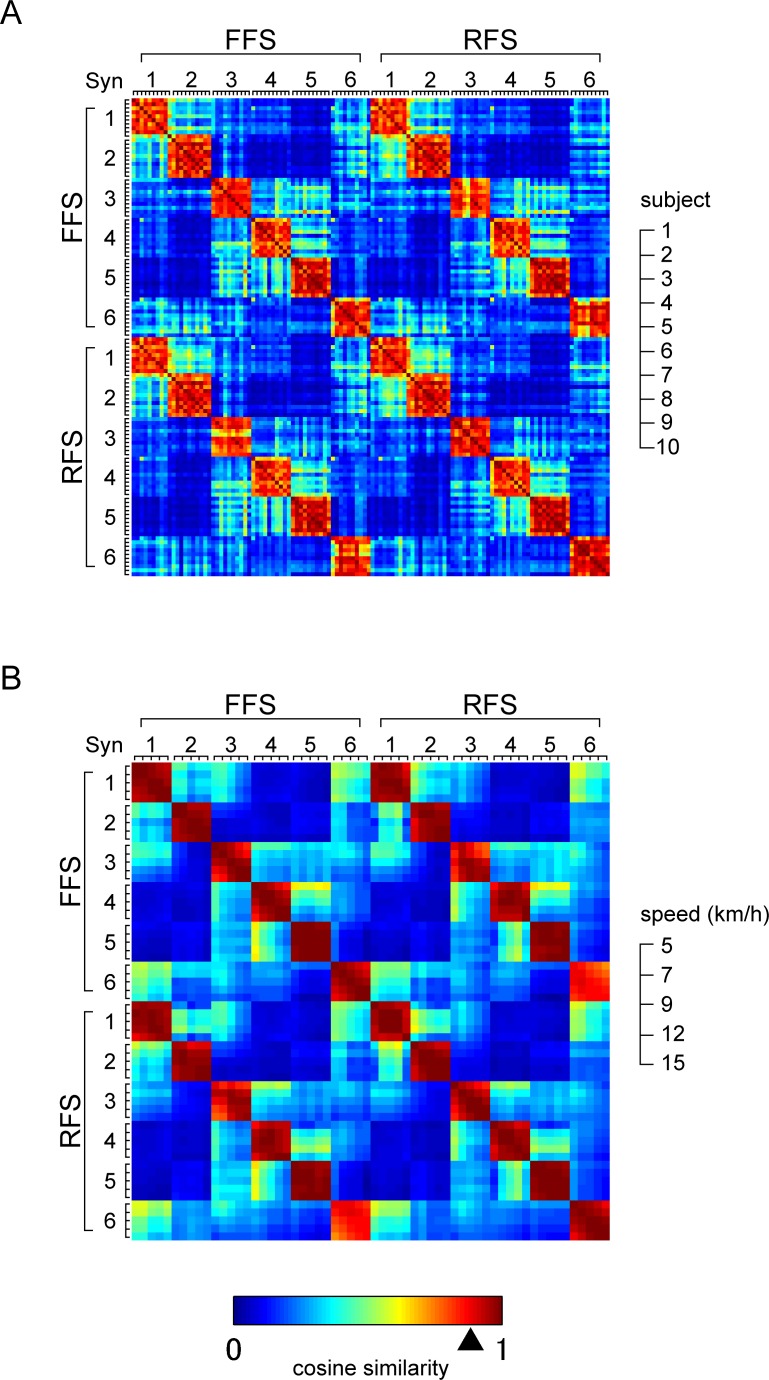
Cosine similarity between muscle synergies. (A) Cosine similarity between all possible combinations of muscle synergies extracted from running at 15 km/h. (B) Cosine similarity between all possible combinations of muscle synergies extracted from Sub2. The arrowhead below the color bar indicates significant similarity value (*r* = 0.868, *p* = 0.05).

Six muscle synergies were extracted for all conditions using NMF. We examined the similarity in weightings of muscle synergies using a cosine similarity analysis (*p* < 0.05 when *r* > 0.868). Fig 4A shows the cosine similarity values expressed as a color map between all possible combinations of muscle synergies extracted from running at 15 km/h. The six muscle synergies (Syn1-6) were quite similar across subjects in both FFS and RFS (mean ± SD across subjects and synergies: FFS, *r* = 0.820 ± 0.104; RFS, *r* = 0.844 ± 0.088). These characteristics were also observed in muscle synergies extracted from running at the other speeds. As shown in Fig 4B, the similarity in muscle synergies across speeds was also high (mean ± SD across speeds and synergies: FFS, *r* = 0.958 ± 0.038; RFS, *r* = 0.958 ± 0.036), which represents the cosine similarity values between all possible combinations of muscle synergies extracted from Sub2. The six muscle synergies were also quite similar between FFS and RFS (mean ± SD across speeds and synergies: *r* = 0.927 ± 0.038), but the similarity in Syn3 and Syn6 between FFS and RFS was relatively low (mean ± SD across speeds: Syn3, *r* = 0.873 ± 0.070; Syn6, *r* = 0.807 ± 0.043) compared to Syn1, Syn2, Syn4, and Syn5 (mean ± SD across speeds: Syn1, *r* = 0.959 ± 0.034; Syn2, *r* = 0.974 ± 0.016; Syn4, *r* = 0.963 ± 0.031; Syn5, *r* = 0.985 ± 0.006). These characteristics of muscle synergies across speeds were common to all subjects.

### General characteristics of muscle synergies

**Fig 5:**
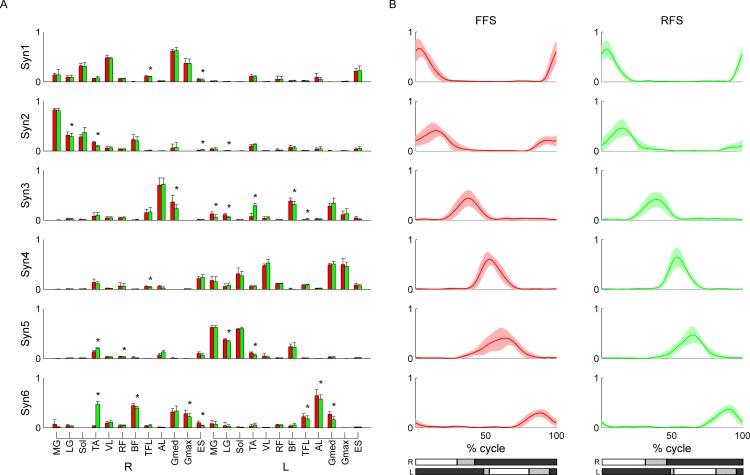
Typical example of muscle synergies extracted from FFS and RFS. (A) The weightings for muscles in muscles synergies (mean over speeds ± SD). (B) The time-course activation patterns of muscles synergies in one cycle (mean over cycles and speeds ± SD). The red and green represent FFS and RFS, respectively. Asterisks represent significant differences at *p* < 0.05. Bars at the bottom of activation patterns represent the stance and swing phases in one cycle (white: stance phase, gray: swing phase, shaded: mean ± SD of liftoff or touchdown timing over cycles and speeds).

A typical example of the six muscle synergies is shown in Fig 5. Syn1 was activated at the time of the right foot touchdown, and it mainly recruited the right VL, Gmed, and Gmax. Syn1 absorbed the touchdown impact and stabilized joints. Syn2 was activated during the time between the right foot touchdown and right foot liftoff or during the right leg stance phase, and it mainly recruited the right plantar flexors (MG, LG, and Sol) and BF. Syn2 pushed off from the ground with plantar flexion of the ankle joint and extension of the hip joint. Syn3 was activated during the time between the right foot liftoff and left foot touchdown, and it mainly recruited the right TFL and AL, along with the left BF. Syn3 lifted the right leg with flexion of the right hip joint and moved the left leg down with extension of the left hip joint. Similarly, Syn4 was activated at the time of the left foot touchdown and absorbed the touchdown impact. Syn5 was activated during the left leg stance phase and pushed off from the ground. Syn6 was activated during time between the left foot liftoff and right foot touchdown and lifted the left leg and moved the right leg down.

These characteristics were common to both FFS and RFS for all subjects. However, several muscle weightings were significantly different for each foot strike pattern (*p* < 0.05). In particular, the weightings of the left TA in Syn3 and right TA in Syn6 showed a large difference between FFS and RFS, and this difference was observed in all subjects except Sub7. Sub7 did not show significant difference in the weightings of TA between FFS and RFS.

### Phase shifts in activation patterns

**Fig 6:**
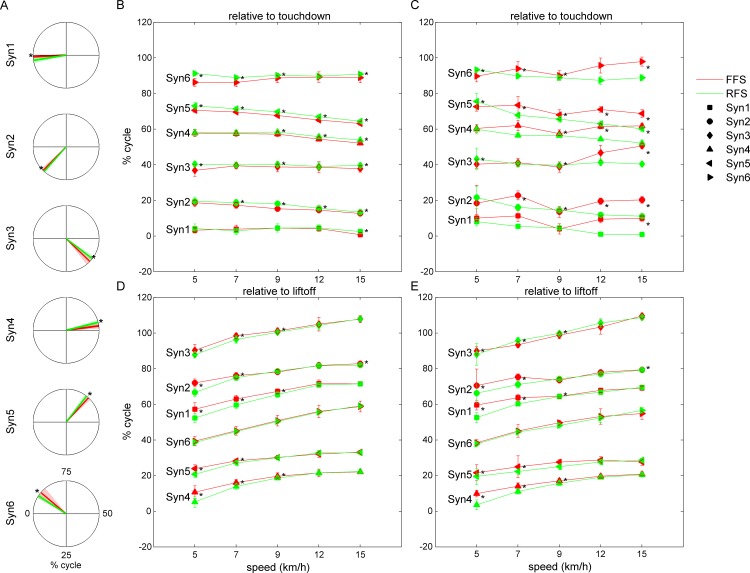
Timing of the main peaks of muscle synergy activity. (A) The example of the circular data for the main peak timings (Sub1 at 15 km/h). (B) Phase delay in the activity of RFS muscle synergies relative to the right foot touchdown for Sub1. (C) Phase delay in the activity of FFS muscle synergies relative to the right foot touchdown for Sub5. (D) Phase shifts relative to the right foot liftoff for Sub1. (E) Phase shifts relative to the right foot liftoff for Sub5. Asterisks represent significant differences at *p* < 0.05.

To compare the activation patterns of muscle synergies extracted from FFS and RFS, we first examined the phase shifts of muscle synergy activity. To quantify the phase shifts, the timing of the main peaks of the muscle synergy activity in one cycle was calculated and transformed to radians (Fig 6A). Eight out of ten subjects (Sub1-3 and Sub6-10) showed the tendency of a phase delay in the activity of the RFS muscle synergies (Fig 6B). In contrast, the other subjects (Sub4-5) showed the tendency of a phase delay in the activity of the FFS muscle synergies (Fig 6C). However, when we defined the gait cycle with respect to right foot liftoff instead of right foot touchdown, all subjects showed a phase delay in the activity of the FFS muscle synergies, especially at lower speeds, and almost no phase shift between FFS and RFS at higher speeds (Fig 6D and 6E). Examining the change in the main peak timing with an increase in speed, in both FFS and RFS, the main peak of the activity tended to show a slight phase advance relative to the foot touchdown, and in contrast, the phase delay relative to foot liftoff.

### Difference in duration of activation patterns

**Fig 7:**
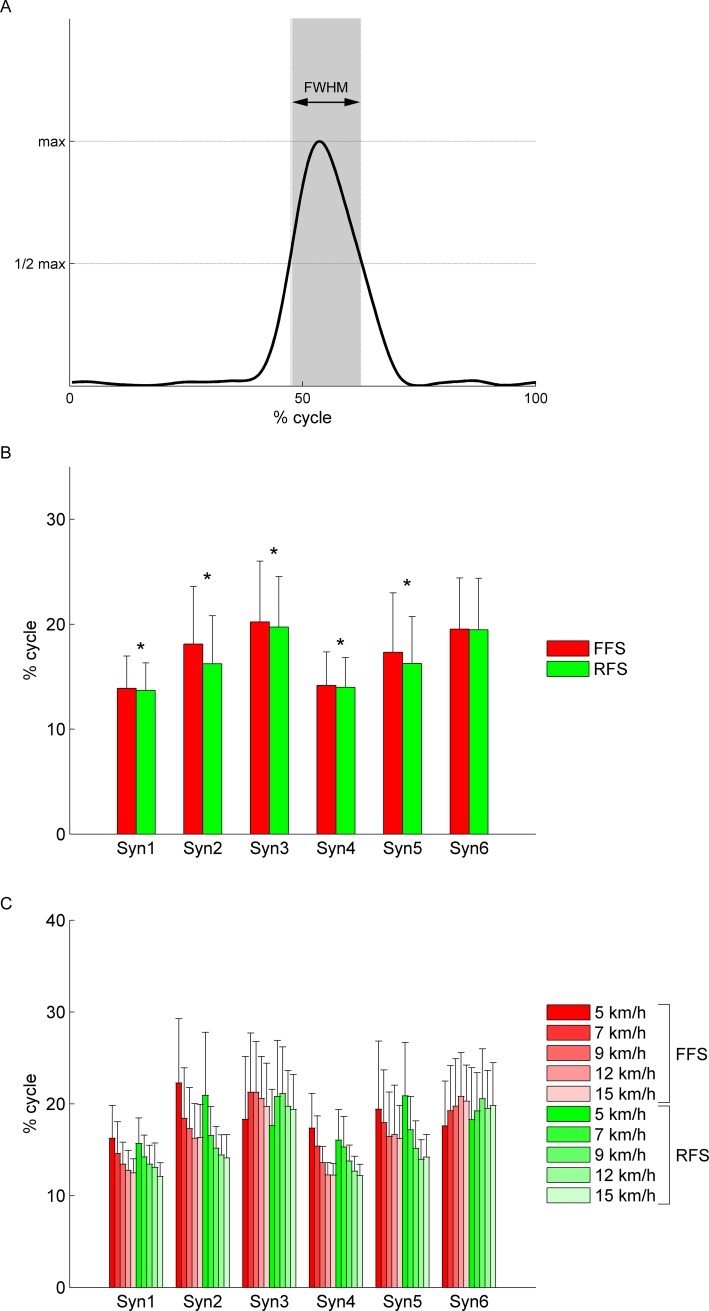
Full-width at half-maximum (FWHM) of the main peak activity. (A) The explanatory drawing of the FWHM of muscle synergy activity. (B) The FWHM of the main peak activity of muscle synergies (mean over cycles, speeds, and subjects ± SD). (C) The FWHM of the main peak activity of muscle synergies at all speeds (mean over cycles and speeds ± SD).

We compared the activation patterns of muscle synergies in terms of the duration of the activity. To estimate the duration, we calculated the FWHM of the main peak (Fig 7A) [17,22]. Fig 7B shows the FWHM of both FFS and RFS muscle synergies (mean over cycles, speeds, and subjects ± SD). All of six synergies showed longer durations of activity in FFS. Syn1-5 showed significant difference (*p* < 0.05). Especially, Syn2 and Syn5, which mainly recruited the plantar flexors of the stance leg, showed clearly longer durations of activity in FFS (*p* < 0.001).

We also examined the changes in the FWHM with an increase in speed. Fig 7C shows the FWHM of both FFS and RFS muscle synergies at all speeds (mean over cycles and subjects ± SD). The FWHM of Syn1-2 and Syn4-5 decreased with an increase in speed. On the other hand, the FWHM of Syn3 and Syn6 tended to increase with an increase in speed. Because the proportion of stance phase to one cycle decreased and that of the swing phase increased with an increase in speed, the changes in the FWHM seemed to correspond to the changes in the duration of the stance and swing phases.

### Difference in magnitude of activation patterns

**Fig 8:**
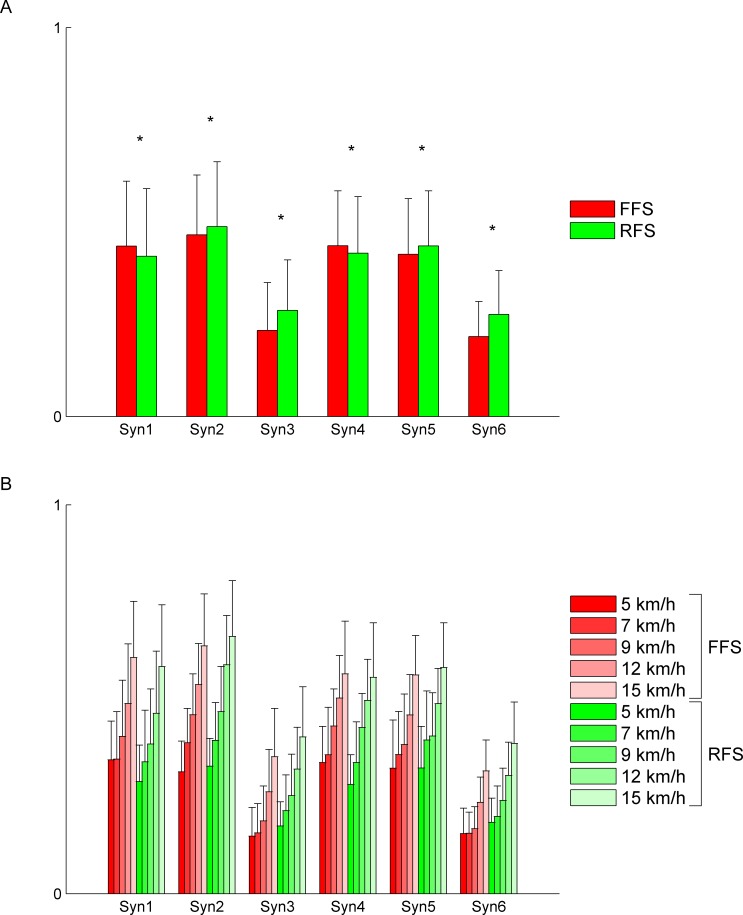
Root mean square (RMS) of the muscle synergy activity during the FWHM. (A) The RMS of the activity of muscle synergies (mean over cycles, speeds, and subjects ± SD). Asterisks represent significant differences at *p* < 0.05. (B) The RMS of the activity of muscle synergies at all speeds (mean over cycles, and subjects ± SD).

We examined the differences in magnitudes among the activation patterns. The magnitude of muscle synergy activity was defined as the RMS of the activity during the FWHM. Fig 8A shows the RMS of the main peak activity of both FFS and RFS muscle synergies (mean over cycles, speeds, and subjects ± SD). Syn2-3 and Syn5-6 showed larger magnitudes in RFS, whereas Syn1 and Syn4 showed larger magnitudes in FFS. All of six synergies showed significant differences between FFS and RFS (*p* < 0.05).

We also examined the changes in the RMS with an increase in speed. Fig 8B shows the RMS of the main peak activity of muscle synergies at all speeds (mean over cycles and subjects ± SD). All six synergies in both FFS and RFS showed larger magnitudes with an increase in speed.

## Discussion

We examined muscle activation over a range of speeds during running with both FFS and RFS. The EMG activity obtained in this study (Fig 3) showed characteristics similar to those observed in previous studies that compared muscle activity between FFS and RFS running [4,5]. These characteristics included increased activity in TA during the terminal swing phase in RFS and increased activity in the plantar flexors during the terminal swing phase in FFS. Using NMF, six muscle synergies were extracted in all conditions (Fig 5). The six synergies showed sequential activity in a cycle, and each synergy had a specific function. Muscle synergies that showed separate peaks in activity during human locomotion, such as walking and running, were also observed in previous studies [15,17,18,22,23]. The recruitment of muscles in each synergy was similar across subjects (Fig 4A) and also similar across speeds (Fig 4B). These results suggest that the same six synergies are adopted across speeds and that a basic pattern of coordinated muscle activity is common to all subjects during running. The muscle synergies extracted from FFS and RFS running were also similar to each other. However, Syn3 and Syn6 showed relatively lower similarity values between FFS and RFS (Fig 4B). When comparing the weightings of muscle synergies, some muscle weightings showed significant differences between FFS and RFS (Fig 5A). In particular, the significant differences in the weightings for TA before the touchdown in Syn3 and Syn6 were large (*p* < 0.003 for Sub2). These large differences in weightings may have resulted in the relatively lower similarity values of Syn3 and Syn6 between FFS and RFS. Differences between FFS and RFS were also found in the activation patterns in terms of the phase shift for the timing of the peak activity as well as the duration and magnitude of the peak activity.

### Differences in muscle synergies between FFS and RFS

The main purpose of this study was to compare muscle synergies for FFS running with those for RFS running. The weightings for the muscle synergies were similar between FFS and RFS, even though some muscle weightings showed significant differences (*p* < 0.05). The weightings for TA before the touchdown in Syn3 and Syn6 showed especially large differences that were common to all subjects except Sub7. These differences correspond to differences in the EMG activity, which included increased activity in TA during the terminal swing phase in RFS. The differences in the weightings of TA make it possible to dorsiflex the ankle joint just before the touchdown in RFS, thus allowing the heel to land first [1,2,4–7].

We also compared the activation patterns of muscle synergies. To examine the phase shifts of the muscle synergy activity, we focused on the timing of the main peak in activity. When the gait cycle was defined by successive right foot touchdown, eight out of ten subjects (Sub1-3 and Sub6-10) showed a phase delay in RFS muscle synergy activity (Fig 6B), whereas the other subjects (Sub4-5) showed a phase delay in FFS muscle synergy activity (Fig 6C). In contrast, all subjects showed a phase delay in FFS muscle synergy activity, especially at lower speeds and with almost no phase shift at higher speeds when the gait cycle was defined by successive right foot liftoff (Fig 6D and 6E). Cappellini et al. [17] also reported differences in phase shift patterns between the timing of the peak activity relative to the foot touchdown and the timing of the peak activity relative to the foot liftoff when comparing the temporal components extracted from walking and running. In the previous study [17], a specific temporal component showed unique phase shifts unlike the phase shifts of the other temporal components. However, in the present study, all six synergies showed similar phase shifts within the same subjects. Thus, at least in the case of controlling foot strike patterns during running, it seems that the activation timings of all muscle synergies adopted are uniformly modulated and that the timings are controlled with reference to foot liftoff rather than foot touchdown.

When comparing the duration of the muscle synergy activity assessed by the FWHM of the main peak (Fig 7A), we found that Syn2 and Syn5, which mainly recruited the plantar flexors in the stance leg, showed a highly significantly longer (*p* < 0.001) duration of activity in FFS (Fig 7B). This difference in duration corresponds to the difference in the EMG activity, which included increased activity in the plantar flexors during the terminal swing phase in FFS or an earlier onset of plantar flexor activity. This allows plantar flexion of the ankle joint before touchdown [1,2,4–7] and the ball of foot to land first in FFS and absorb the touchdown impact using the plantar flexors with larger ankle planter flexion moments [2,8,27] and Achilles tendon forces [2]. This absorption results in a relatively mild vertical ground reaction force at the touchdown in FFS [1,2,4,27]. When we looked at the FWHM in each subject, we found that only in Sub1, Syn2 and Syn5 showed significantly shorter durations of activity (*p* < 0.05) in FFS. However, instead of the shorter activity duration of Syn2 and Syn5 in FFS, the weightings of the plantar flexors when the leg landed in Syn1 and Syn4 were significantly larger in FFS in Sub1. However, in the other subjects, Syn1 and Syn4 did not necessarily have larger weightings for the plantar flexors in FFS. As for Sub1, this difference in weightings corresponds to the larger activity of the plantar flexors during the terminal swing phase in FFS.

We also examined the differences in magnitudes of muscle synergy activity defined by the RMS of the activity during the FWHM. We found that Syn2-3 and Syn5-6 showed significantly larger magnitudes in RFS and that Syn1 and Syn4 showed significantly larger magnitudes in FFS (*p* < 0.05) (Fig 8A). The increased activity of Syn1 and Syn4 in FFS may contribute to more effective absorption of the touchdown impact. The increased activity of Syn2 and Syn5 in RFS seems to originate from the necessity for plantar flexion of the ankle joint to occur after the heel strike [2,8]. The increased activity of Syn3 and Syn6 in RFS seems to be related to the necessity for dorsal flexion of the ankle joint to occur before touchdown [1,2,4–7], as is the case for the weightings.

The present results indicated the activation patterns of muscle synergies also showed some changes with an increase in speed (Figs 6, 7C and 8B). However, there was no determinant difference between FFS and RFS in how the activation patterns change with an increase in speed.

In the present study, Sub5 and Sub10 were habitual FFS runners and the other subjects were habitual RFS runners. However, there was no distinct difference between habitual FFS and RFS runners about the composition and activation profile of muscle synergies. This result suggests the possibility that the differences we found in the present study between FFS and RFS would not come from the experiences of individuals but be inherent in individuals.

### Neural control for human running

In this study, we extracted six muscle synergies during FFS and RFS running. In previous studies, five temporal components [17] or approximately nine muscle synergies [18] were extracted to explain EMG activity during human running. The differences in the number of synergies between the present study and those reported in previous studies seems to result from a difference in the number of muscles recorded, the extraction method (principal component analysis in Cappellini et al. [17]), or the VAF threshold criterion (overall VAF > 95% and each muscle VAF > 80% in Hagio et al. [18]).

The six muscle synergies extracted during FFS and RFS were similar. However, we found some differences in the muscle weightings and activation patterns. Differences in activation patterns, such as phase shifts and changes in the duration and magnitude, were also observed in previous studies with humans and cats that performed walk-to-run or run-to-walk transitions [17,18] and stepping over various obstacles [28]. The changes in activation patterns are easy to understand because they could be accomplished by controlling the timing, duration, and magnitude of the descending signals from the CNS. Therefore, the changes in the activation patterns between FFS and RFS, and also the changes with an increase in speed observed in this study, seems to reflect the changes in the timing, duration, and magnitude of the descending signals from the CNS. However, the difference in muscle weightings is difficult to understand because the origin of the muscle synergies would include the complexity in the neural circuitry.

By comparing the muscle weightings of muscle synergies between FFS and RFS, we found several significant differences in muscle weightings (*p* < 0.05) (Fig 5A). In particular, the differences in the weightings for TA before the touchdown in Syn3 and Syn6 were large and common to all subjects except Sub7. To explain these differences in weightings for TA, we suggest two possibilities regarding the neural control mechanisms with reference to the possible corticospinal connections proposed by Krouchev and Drew [28]. Given that a single subpopulation of corticospinal tracts connect with spinal interneurons that project to the motoneurons of all muscles in a synergy, (1) the CNS send signals to entirely different synergies for each foot strike pattern. That is, Syn3 and Syn6 in FFS and those in RFS are entirely different. Alternatively, given that there are several subpopulations of corticospinal tracts that are linked by intercortical connections and discharge simultaneously during running that connect with different populations of spinal interneurons, and each of these interneuron populations innervate only part of the total muscle synergy, that is, a synergy is composed of several subsets, (2) a subset exists in Syn3 and Syn6 that recruit only TA or TA and other muscles, and the CNS selectively activates the subsets.

We cannot say which of these two explanations are correct, but the fact that some muscle weightings, including those for TA, differ significantly for each foot strike pattern suggests that there are more than six synergies or several subsets in the six synergies. In recent studies [28–32], muscle synergies were extracted by focusing on the onset and offset timing of EMG activity. To more precisely reveal the neural control mechanisms underlying human running, more detailed analyses are needed.

## Conclusions

Six muscle synergies were extracted from FFS and RFS, and these muscle synergies were similar between FFS and RFS, even though some muscle weightings in the synergies showed significant differences between FFS and RFS. The activation patterns of muscle synergies were also different in terms of their timing, duration and magnitude of the main peak activity. These results suggest that human running is accomplished by using six muscle synergies and that a foot strike pattern is controlled by the change in the timing, duration, and magnitude of the activation patterns of the synergies. The differences in muscle weightings between FFS and RFS suggest the existence of other muscle synergies or subsets of muscle synergies. However, the six synergies extracted in this study could account for the EMG activity during both FFS and RFS running of all subjects and captured the basic muscle coordination pattern underlying human running.
